# Antimicrobial and Antibiofilm Activities of Highly Soluble Polypyrrole Combined with Benzoic Acid against Multidrug-Resistant *Klebsiella pneumoniae*

**DOI:** 10.1021/acsomega.5c08466

**Published:** 2025-11-24

**Authors:** Danillo S. Rosa, Brendo A. dos S. da Cruz, Nayara Andreo, Betilde de M. Silva, Priscila I. de Figueirêdo, Fernando A. G. da Silva Júnior, Helinando P. de Oliveira, Flavia F. Aburjaile, Carine R. Naue, Mateus M. da Costa

**Affiliations:** † 67744 Universidade Federal Rural de Pernambuco, Rua Dom Manuel de Medeiros, s/n, Dois Irmãos, Recife, Pernambuco 52171 900, Brazil; ‡ Universidade Federal do Vale do São Francisco (UNIVASF), Avenida José de Sá Maniçoba, s/n, Centro, Petrolina, Pernambuco 56300-000, Brazil; § 28114 Universidade Federal de Minas Gerais, Avenida Presidente Antonio Carlos, 6627, Pampulha, Belo Horizonte, Minas Gerais 31270-901, Brazil; ∥ 700234 Hospital Universitário da Universidade Federal do Vale do São Francisco, Avenida José de Sá Maniçoba, s/n, Centro, Petrolina, Pernambuco 56304-205, Brazil

## Abstract

Multidrug-resistant *Klebsiella pneumoniae* represents a critical global public health threat, with limited therapeutic options available due to its biofilm-forming capacity. This study investigated the antimicrobial and antibiofilm potential of benzoic acid (BA), an organic acid, and highly soluble polypyrrole (Hs-PPy), a synthetic polymer, against *K. pneumoniae* isolates. Forty-seven clinical isolates from infected/colonized patients and one reference strain were evaluated. Antimicrobial susceptibility was determined for 16 antibiotics. Antimicrobial activity was assessed via broth microdilution against 25/47 isolates and the reference strain. Biofilm formation and substance interference were evaluated using crystal violet staining. All isolates demonstrated multidrug resistance, with >91% showing resistance to ampicillin, cefepime, ceftriaxone, levofloxacin, ertapenem, piperacillin-tazobactam, ampicillin-sulbactam, cefoxitin, ciprofloxacin, meropenem, imipenem, and trimethoprim-sulfamethoxazole, while 57.44% were resistant to polymyxin B. Susceptibility to amikacin and gentamicin was observed in 95.74% and 51.06% of isolates, respectively. Hs-PPy showed no intrinsic antimicrobial activity, whereas BA exhibited inhibitory and bactericidal effects at 2–4 mg/mL. Among biofilm producers, one isolate demonstrated strong formation, while others showed weak production. The Hs-PPy/BA combination effectively inhibited biofilm formation in the strong producer. These findings suggest that Hs-PPy/BA combination therapy offers significant advantages for controlling biofilm formation in multidrug-resistant *K. pneumoniae* infections.

## Graphical abstract

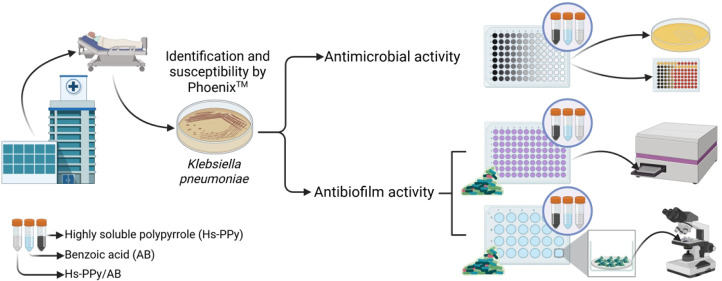

## 1 Introduction

Healthcare-associated infections (HAIs) represent a significant cause of morbidity and mortality worldwide, constituting a major public health concern.^1^ Both in Brazil and globally, *Klebsiella* spp. rank among the most prevalent pathogenic microorganisms associated with HAIs.^2,3^ However, although these bacteria are part of the natural intestinal microbiota in animals, including humans,^4^ they can cause various infections under dysbiotic conditions.^5^

*Klebsiella pneumoniae* is a Gram-negative bacterium of the *Enterobacteriaceae* family, widely distributed in both hospital and community settings.^6^ This pathogen is responsible for diverse infections, including necrotizing soft tissue infections, pneumonia, pyogenic liver abscesses, bloodstream infections, meningitis, endophthalmitis, and urinary tract infections, with mortality rates ranging from 25 to 70%.^7^

These concerns are exacerbated by the rise of antimicrobial resistance, as multidrug-resistant pathogens pose one of the greatest global health challenges and a serious public health threat.^8^ Furthermore, *K. pneumoniae* must overcome multiple mechanical and chemical barriers to establish infection while evading the humoral and cellular immune defenses of the host.^9^ A key virulence mechanism is biofilm formation,^10^ which significantly increases treatment difficulty compared to infections caused by nonbiofilm-forming strains.^11^

Biofilms not only facilitate microbial dissemination but also contribute to increased morbidity, mortality, and healthcare costs.^12^ Given the strong association between HAIs and the spread of infectious agents, key preventive measures include: hand hygiene, maintaining a safe and hygienic hospital environment, patient cohorting, public health surveillance, appropriate antibiotic use, and adherence to patient safety guidelines.^13^

Researchers have investigated new compounds capable of eliminating multidrug-resistant bacteria, including benzoic acid (BA—C_6_H_5_COOH), an organic acid that has emerged as an alternative compound in the search for new drugs and has shown notable antimicrobial activity.^14–16^ Additionally, synthetic polymers have also demonstrated antimicrobial and antibiofilm activity, both alone and in combination, as is the case with highly soluble polypyrrole (Hs-PPy—[C_6_H_10_N_2_]_n_),^17^ which exhibits activity against bacteria and yeasts.^18^

From this perspective, this study aimed to (i) evaluate the susceptibility profile of clinical *K. pneumoniae* isolates from the University Hospital of the Federal University of Vale do São Francisco (HU-UNIVASF), Petrolina, Pernambuco, Brazil; (ii) assess the antibacterial action of Hs-PPy alone and in combination with benzoic acid; (iii) investigate the biofilm-forming capacity of these *K. pneumoniae* isolates; and (iv) evaluate the antibiofilm potential of Hs-PPy alone and in combination with benzoic acid.

## 2 Materials and Methods

### 2.1 Bacterial Isolates

A total of 47 *K. pneumoniae* isolates were obtained from patients colonized and/or infected with polymyxin B-resistant or -susceptible strains at the University Hospital of the Federal University of Vale do São Francisco (HU-UNIVASF), Petrolina, Pernambuco, Brazil, between July and November 2021 (Table S1). The isolates originated from the following sources: blood culture (2/47), rectal swab (18/47), surveillance culture (6/47), tracheal secretion (12/47), urine culture (6/47), brain abscess capsule (1/47), soft tissue (1/47), and urethral swab (1/47). All isolates are registered in the National System for the Management of Genetic Heritage (SisGen, No. A01C760) and were identified using the automated Phoenix system (Becton Dickinson, Franklin Lakes, USA), which was also used to assess antimicrobial susceptibility. These results were classified as sensitive, intermediate, and resistant according to the methodology of the Clinical and Laboratory Standards Institute (CLSI)^19^ and the multiple resistance index (MRI) was calculated according to Krumperman.^20^ The study was approved by the Ethics Committee of *Universidade da Região da Campanha* (URCAMP) under protocol No. 5.079.225. The reference strain *K. pneumoniae* subsp. *pneumoniae* ATCC 13883 (American Type Culture Collection, ATCC, Manassas, USA) was included in the assays. All samples were stored at −20 °C in Brain Heart Infusion (BHI, KASVI, São José dos Pinhais, Brazil) supplemented with 40% glycerol. Prior to testing, isolates were cultured on Tryptic Soy Agar (TSA, KASVI) at 37 °C for 24 h. For antimicrobial testing, 25 isolates were randomly selected. Additionally, *Staphylococcus aureus* ATCC 25923 was used as a positive control for biofilm formation.

### 2.2 Antimicrobial Activity

#### 2.2.1 Polymyxin B Susceptibility Testing Using the POLICIMBAC Commercial Kit

The commercial POLIMBAC kit (PROBAC DO BRASIL, São Paulo, Brazil) was used to assess polymyxin B susceptibility. The kit contains dehydrated, cation-adjusted Mueller Hinton Broth (MHB, KASVI) with decreasing concentrations of polymyxin B (64–0.125 μg/mL). First, 100 μL of a bacterial suspension in saline solution (10^5^ CFU/mL) was added to each well, followed by incubation at 37 °C for 24 h. Results were interpreted according to the guidelines of the manufacturer.

#### 2.2.2 Preparation of Antimicrobial Solutions

Hs-PPy was synthesized at 2 mg/mL via chemical oxidation as described by Da Silva Júnior et al.^18^ A stock solution of benzoic acid (BA, 8 mg/mL; Proquímios, Rio de Janeiro, Brazil) was prepared in 10% dimethyl sulfoxide (DMSO, NEON, São Paulo, Brazil) and 90% sterile distilled water.

The Hs-PPy/BA composite was prepared according to Kang et al.^21^ Briefly, 1.08 g sodium dodecyl sulfate (SDS) was dissolved in 100 mL ultrapure water, followed by addition of 500 μL pyrrole (Py) under constant stirring for 45 min. BA was then incorporated at a final concentration of 8 mg/mL. Polymerization was initiated by dropwise addition of 50 mL aqueous ammonium persulfate (APS, 5.12 mg/mL) to the mixture, with continuous stirring for 35 min. The solution was stirred for an additional 1 h, yielding a black suspension that was stored at 4 °C for 24 h. The final solution was composed of 2 mg/mL of Hs-PPy with 8 mg/mL of BA.

#### 2.2.3 Broth Microdilution Assay for Antimicrobial Activity

The minimum inhibitory concentration (MIC) and minimum bactericidal concentration (MBC) of Hs-PPy and BA (alone and combined) were determined by broth microdilution.^22^ Serial dilutions (1:2) of stock solutions were prepared in 96-well microplates (OLEN, São José dos Pinhais, Brazil) containing Mueller-Hinton broth (MHB, KASVI) to a final volume of 100 μL. Bacterial suspensions (∼1.5 × 10^6^ CFU/mL) were inoculated (10 μL/well), including growth controls. Sterility controls contained MHB only. A control diluent, containing serial dilutions from the 10% DMSO stock, was included to assess whether it exhibits antimicrobial activity and interferes with the BA results. Plates were incubated at 37 °C for 24 h. For MBC determination, 10 μL from each well was subcultured on Mueller-Hinton agar and incubated at 37 °C for 24 h. Bacterial growth was assessed visually. After MIC plate incubation, 30 μL of 1% 2,3,5-triphenyltetrazolium chloride (TTC, Dinâmica, Indaiatuba, Brazil) was added to each well, followed by 1 h incubation. MIC end points were defined by pink color development. All assays were performed with technical and biological triplicates.

### 2.3 Biofilm Production Analysis and Substance Interference

#### 2.3.1 Biofilm Quantification

Phenotypic quantification of biofilm production was performed using the crystal violet assay (adapted from Stepanović et al.,^23^ and Merino et al.,^24^). Briefly, 5 μL of bacterial suspension (6 × 10^6^ CFU/mL) was added to 96-well microplates (OLEN) containing 195 μL Trypticase Soy Broth (TSB, KASVI). After 24 h incubation at 37 °C, plates were washed three times with 200 μL sterile water and air-dried for 5 min. Biofilms were fixed with 150 μL methanol (ISOFAR, Trenthorst, Germany) for 20 min, then stained with 100 μL 0.25% crystal violet (Proquímios) for 5 min. Following repeated washing, bound dye was solubilized with 200 μL ethanol-acetone (80:20) [99.8% absolute ethanol (NEON) + 99.5% acetone (Fmaia, Belo Horizonte, Brazil)]. Absorbance was measured at 620 nm using an EXPERT PLUS-UV microplate reader (ASYS, Cambridge, U.K.). All samples were analyzed in technical triplicates across three independent days. Biofilm production was classified qualitatively according to Stepanović et al.^25^ *S. aureus* ATCC 25923 was included as a biofilm-positive control in TSB supplemented with 0.25% glucose (TSBg).

#### 2.3.2 Interference of PPy, BA, and Hs-PPy/BA on Biofilm Formation

The inhibitory effects of PPy, BA, and Hs-PPy/BA on biofilm formation were evaluated using an adapted protocol.^24,26^ Bacterial suspensions at 3 × 10^5^ CFU/mL in TSB were incubated with test substances at 12 MIC concentrations in 96-well plates (100 μL/well). Negative controls contained TSB only, with substance-only controls for each concentration. Washing, methanol fixation, crystal violet staining, and ethanol-acetone resolubilization followed the same protocol as the quantification assay.

#### 2.3.3 Light Microscopy Analysis of Biofilm Interference

The biofilm analysis assay was performed according to the methodology adapted from Wang et al.,^27^ using isolate 5825, which demonstrated strong biofilm formation. Glass coverslips (15 mm diameter × 0.2 mm thickness) were placed in wells of a 24-well plate containing: for the positive control, 1 mL of bacterial suspension at 1.5 × 10^5^ CFU/mL in TSB; for the treatments, 1 mL of bacterial suspension at 1.5 × 10^5^ CFU/mL in TSB containing final concentrations of 1/2 and 1/4 MIC of the test substances; and the negative control, containing 1 mL of TSB. The plate was incubated at 37 °C for 24 h. After this period, the wells were washed with distilled water, and the biofilm was fixed with 1 mL of analytical grade methanol (ISOFAR) for 20 min and dried overnight (adapted from Wang et al.,^27^). Subsequently, 100 μL of 0.25% crystal violet was added for 5 min. Finally, the coverslips were washed three times with distilled water, removed, and observed under an optical microscope (B1 series, Motic, China).

### 2.4 Statistical Analysis

The biofilm interference results were analyzed using parametric multiple comparisons tests, specifically Two-way ANOVA with the posthoc test of Tukey. Statistical analyses were performed using GraphPad Prism 8 software, and results were plotted as mean ± standard deviation. For all tests, a *p*-value <0.05 was considered statistically significant.

## 3 Results

### 3.1 Susceptibility Profile

Based on results obtained from the Phoenix platform, all *K. pneumoniae* isolates were characterized as multidrug-resistant (MDR) (Figure 1). These isolates were also classified as high-risk to public health, exhibiting multiple resistance index (MRI) values ranging from 0.37 to 0.87 (MRI > 0.2).

**Figure 1:**
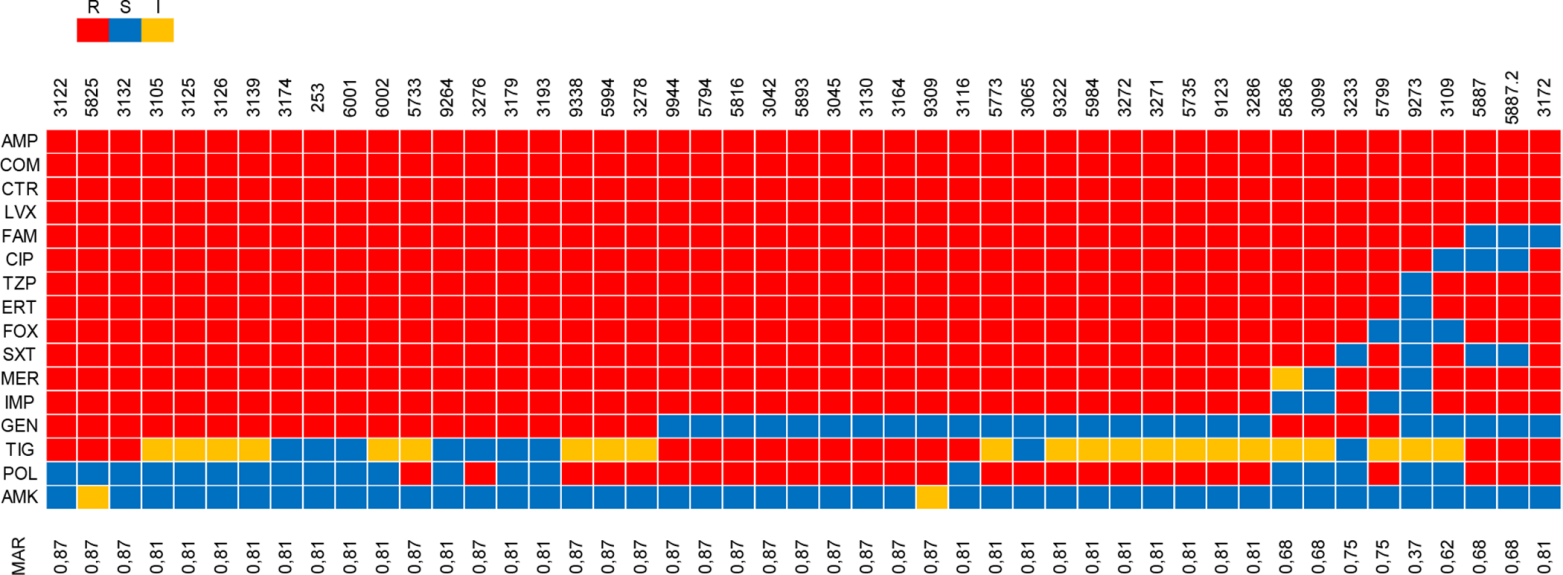
Heatmap representing the multiple antibiotic resistance index (MRI) of *Klebsiella pneumoniae* isolates. Rows represent antimicrobial agents and columns represent bacterial isolates. Red blocks indicate resistance (R), blue blocks indicate susceptibility (S), and yellow blocks indicate intermediate activity (I). Antimicrobials evaluated by the automated Phoenix system: AMP (ampicillin); AMK (amikacin); CIP (ciprofloxacin); COM (cefepime); CTR (ceftriaxone); ERT (ertapenem); FAM (ampicillin-sulbactam); FOX (cefoxitin); GEN (gentamicin); IMP (imipenem); LVX (levofloxacin); MER (Meropenem); SXT (trimethoprim-sulfamethoxazole); TZP (piperacillin-tazobactam); and TIG (tigecycline). Evaluated by the POLICIMBAC commercial kit: POL (polymyxin B).

Among the 16 antimicrobials tested, all *K. pneumoniae* isolates (100%) were resistant to ampicillin, cefepime, ceftriaxone, and levofloxacin, followed by ertapenem and piperacillin-tazobactam (97.87%; 46/47). High resistance rates were also observed for ampicillin-sulbactam, cefoxitin, ciprofloxacin, and Meropenem (93.62%; 44/47 of isolates), imipenem and trimethoprim-sulfamethoxazole (91.49%; 43/47), and polymyxin B (57.44%; 27/47). Amikacin showed the highest susceptibility rate (95.74%), followed by gentamicin (51.06%; 24/47). For tigecycline, 46.81% (22/47) of isolates were classified as intermediate, 34.04% (16/47) as resistant, and only 19.15% (9/47) as susceptible (Figure 2).

**Figure 2:**
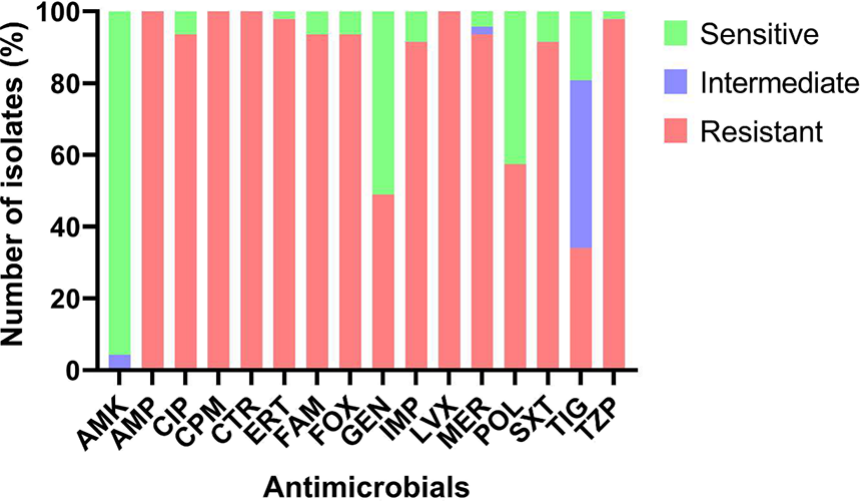
Susceptibility profile of *Klebsiella pneumoniae* isolates. Evaluated by the automated Phoenix system: AMP (ampicillin); AMK (amikacin); FAM (ampicillin-sulbactam); COM (cefepime); FOX (cefoxitin); CTR (ceftriaxone); CIP (ciprofloxacin); ERT (ertapenem); GEN (gentamicin); IMP (imipenem); LVX (levofloxacin); MER (Meropenem); TZP (piperacillin-tazobactam); TIG (tigecycline); and SXT (trimethoprim-sulfamethoxazole). Evaluated by the POLICIMBAC commercial kit: POL (polymyxin B).

### 3.2 Antimicrobial Activities of Hs-PPy, BA, and Hs-PPy/BA

Hs-PPy did not demonstrate intrinsic antimicrobial activity against any of the *K. pneumoniae* isolates at the tested concentrations (Table 1). BA showed antimicrobial activity with minimum inhibitory concentration (MIC) values ranging from 2 (96.15%; 25/26) to 4 mg/mL (3.85%; 1/26), and minimum bactericidal concentration (MBC) values from 2 (46.15%; 12/26) to 4 mg/mL (53.85%; 14,26). The IC50 for BA was 2 mg/mL and IC90 was 4 mg/mL. In combination with BA, Hs-PPy exhibited antimicrobial activity with MIC and MBC values ranging from 0.25 mg/mL (26.92% (7/26) and 15.38% (4/26) of isolates, respectively) to 1 mg/mL (23.07% (6/26) and 46.15% (12/26), respectively). Additionally, the diluent (DMSO) showed no antimicrobial activity at the concentrations tested.

**Table 1:** Antimicrobial Activity of Highly Soluble Polypyrrole (Hs-PPy), Benzoic Acid (BA), and Hs-PPy/BA against Multidrug-Resistant *Klebsiella pneumoniae* Isolates^a^

	**compounds** (mg/mL)					
	**Hs-PPy** ^b^		**BA**		**combination (Hs-PPy/BA)^c^**	
**no. of the isolate**	MIC	MBC	MIC	MBC	MIC	MBC
9944	>1	>1	2	2	5	5
5773	>1	>1	2	4	2.5	5
5794	>1	>1	2	2	5	5
5799	>1	>1	2	4	2.5	5
5813	>1	>1	2	2	5	5
3042	>1	>1	2	2	2.5	5
3065	>1	>1	2	2	2.5	5
5893	>1	>1	2	4	5	5
5887	>1	>1	2	4	2.5	2.5
9322	>1	>1	2	4	5	5
9338	>1	>1	2	4	2.5	2.5
5984	>1	>1	2	4	2.5	2.5
5994	>1	>1	2	4	1.25	2.5
3272	>1	>1	2	2	5	5
3271	>1	>1	2	4	2.5	2.5
5825	>1	>1	2	2	2.5	5
3125	>1	>1	2	2	2.5	2.5
3126	>1	>1	2	2	2.5	5
9273	>1	>1	2	4	2.5	2.5
3139	>1	>1	2	4	1.25	1.25
3174	>1	>1	2	2	1.25	1.25
3233	>1	>1	2	4	2.5	2.5
253	>1	>1	2	2	1.25	1.25
6001	>1	>1	2	4	1.25	1.25
6002	>1	>1	2	2	1.25	2.5
ATCC 13883	>1	>1	4	4	1.25	2.5

a ATCC: American Type Culture Collection; MIC: minimum inhibitory concentration; and MBC: minimum bactericidal concentration. Stock concentrations: Hs-PPy (2 mg/mL); BA (8 mg/mL).

b The stock solution of the combination is composed of 2 mg/mL of Hs-PPy plus 8 mg/mL of BA, expressed as 10 mg/mL. Therefore, the concentrations presented here correspond to 1/5 of Hs-PPy and 4/5 of BA.

c >1 means that there was no MIC and MBC value for the concentrations tested, which may be above the production value.

### 3.3 Biofilm

All *K. pneumoniae* isolates demonstrated biofilm-forming capability in polyethylene microplates at varying levels (weak or strong). Only one isolate (5825; 3.85%; 1/26) was classified as a strong producer (Figure 3), while the remaining isolates (96.15%; 25/26), including ATCC 13883, were weak producers (Figures 3 and Figure S1). The biofilm-positive control strain *S. aureus* ATCC 25923 was confirmed as a strong producer. When testing subinhibitory concentrations ( 12 MIC) of BA, Hs-PPy, and Hs-PPy/BA, no statistically significant difference was observed between treated and untreated weak biofilm producers. However, the strong producer 5825 showed complete biofilm inhibition (classified as nonbiofilm forming) under all treatment conditions, with significant differences compared to untreated controls (*p* < 0.0001). Due to this marked difference, isolate 5825 was selected for subsequent microscopic analysis.

**Figure 3:**
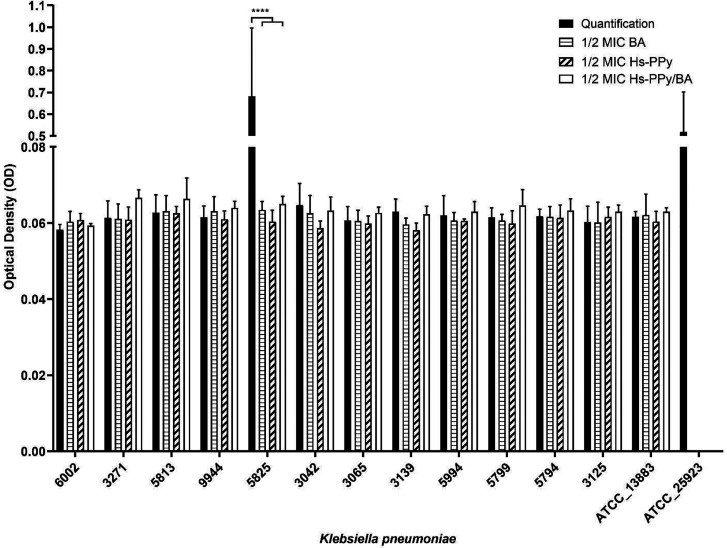
Quantification of biofilm formation and interference by highly soluble polypyrrole (Hs-PPy) and benzoic acid (BA), alone and combined (Hs-PPy/BA), against the biofilm of some *Klebsiella pneumoniae* isolates in the present study. Other isolates can be seen in Figure S1. ATCC_13883: *K. pneumoniae* reference strain; ATCC_25923: methicillin-sensitive *S. aureus* (biofilm-positive control). Negative control: OD620:0.057 ± 0.003 (not subtracted from analyses). ****p < 0.0001.

Light microscopy analysis (Figure 4) revealed that 12 MIC treatments with either compound or their combination significantly reduced 5825 biofilm formation, showing more dispersed cellular arrangements (Figure 4B–D). At 14 MIC concentrations, BA treatment paradoxically enhanced biofilm formation, producing thicker cellular layers than untreated controls (Figure 4G). In contrast, both Hs-PPy and Hs-PPy/BA maintained their biofilm-reducing effects at 14 MIC (Figure 4F,H).

**Figure 4:**
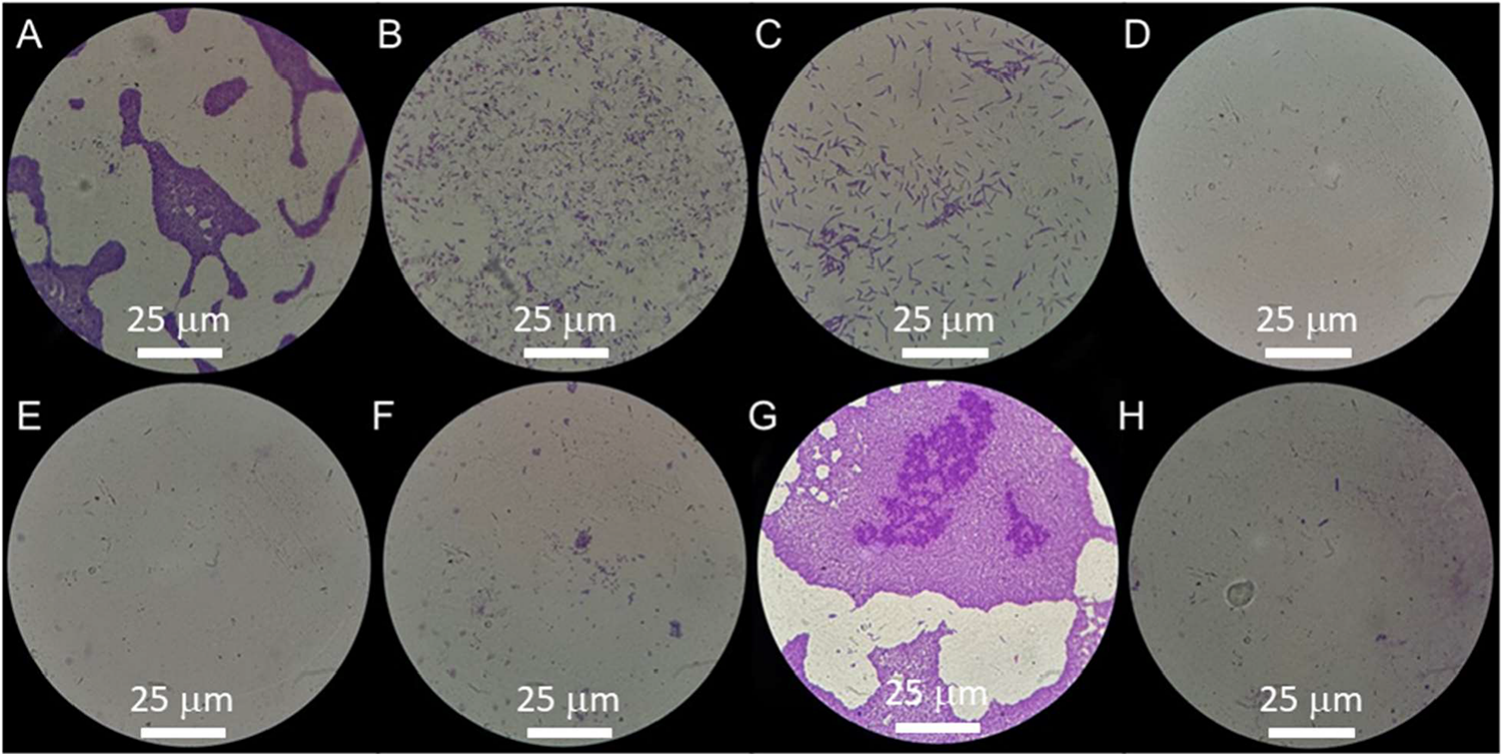
Visual evidence of biofilm formation inhibition in *Klebsiella pneumoniae* 5825 following treatment with (B) 1000 μg/mL of highly soluble polypyrrole (Hs-PPy); (F) 500 μg/mL of Hs-PPy; (C) 1000 μg/mL of benzoic acid (BA); (G) 500 μg/mL of BA; (D) 250/1000 μg/mL of the Hs-PPy/BA combination; and (H) 125/500 μg/mL of Hs-PPy/BA. Light microscopy observations are shown in comparison with positive (A) and negative (E) controls. Reading performed under light microscopy at 1000× magnification.

## 4 Discussion

In the present study, all *K. pneumoniae* isolates were characterized as multidrug-resistant (MDR), revealing a concerning clinical scenario. This pathogen is well-documented as a significant cause of hospital-acquired infections^28^ and is associated with various severe clinical manifestations.^7^ The emergence of MDR *K. pneumoniae* strains is facilitated by the extensive use of antibiotics in hospital settings,^29^ which complicates both treatment and prevention of healthcare-associated infections.^30^ The high multiple antibiotic resistance index (MRI) values observed in these isolates demonstrate substantial selective pressure and confirm the significant risk of resistance dissemination.

Notably, 97.87% of isolates showed resistance to at least one carbapenem antibiotic—the last-resort treatment option for MDR *K. pneumoniae* infections.^31,32^ Carbapenem-resistant Enterobacteriaceae (CRE), including these *K. pneumoniae* isolates, represent an emerging public health crisis due to their resistance to most antimicrobial classes.^33^ This severely limits treatment options for CRE infections, leaving aminoglycosides and polymyxins as primary alternatives.^34^

For 51.06% of isolates in this study, aminoglycosides (amikacin and gentamicin) represented the only therapeutic option based on susceptibility profiles. However, significant concerns exist regarding their clinical use: standard doses often fail to achieve optimal pharmacokinetic (PK) and pharmacodynamic (PD) targets, while carrying risks of nephrotoxicity and rapid resistance development during treatment.^34^ Furthermore, achieving ideal PK/PD parameters proves particularly challenging when the minimum inhibitory concentration (MIC) approaches the susceptibility breakpoint, even with higher dosing regimens.^35^ Despite these limitations, the observed high susceptibility to amikacin suggests it remains an important option for carbapenem-resistant *K. pneumoniae* infections. Importantly, when combined with colistin, amikacin demonstrates increased nephrotoxicity compared to polymyxin B combinations, necessitating cautious use.^36^ These findings underscore the imminent risk of bacterial dissemination within hospital environments.

With regard to Hs-PPy activity, while previous studies demonstrated efficacy against various microorganisms including Gram-negative bacteria,^17,18^ no intrinsic antimicrobial activity was observed against the MDR *K. pneumoniae* clinical isolates in this study. The antimicrobial mechanism of polypyrrole (PPy) typically involves electrostatic interaction between its positive charges and bacterial cell walls, leading to membrane disruption and cytoplasmic content leakage.^37,38^ The lack of activity observed here may be explained by limited Hs-PPy interaction with the cell wall of *K. pneumoniae*, as the polysaccharide capsule—an outer protective layer and key virulence factor—shields the bacterium from host innate immunity.^39,40^ Furthermore, *K. pneumoniae* produces over 79 distinct polysaccharide capsule types,^41,42^ highlighting both its virulence potential and remarkable phenotypic diversity.

Although the polysaccharide capsule serves as an important protective structure, nonencapsulated strains demonstrate greater adhesion capacity to both biotic and abiotic surfaces,^43,44^ suggesting the capsule may actually hinder biofilm formation.^45^ The observed low biofilm production in 96.15% of isolates indicates that capsule formation may contribute to this phenotype. The relationship between capsule formation/type and biofilm production warrants further investigation in isolates exhibiting different capsule types to better understand this virulence mechanism. The complete inhibition of biofilm formation in one isolate by subinhibitory concentrations of Hs-PPy and BA may result from electrostatic interactions between the positive charges of PPy and exopolysaccharide matrix components,^46^ disrupting cell-to-cell adhesion and promoting cellular dispersion.

When combined with 1/2, 1X, or 2X MIC of BA, Hs-PPy demonstrated activity against different isolates. BA and its derivatives have known antimicrobial activity, both alone and in combinations.^47–49^ However, this is the first study to evaluate its action against clinical isolates of MDR *K. pneumoniae*. The activity of BA is explained by its ability to interact with the bacterial cell membrane, penetrate into the intracellular environment, and dissociate at higher pH, releasing charged anions and protons.^15^ Other proposed mechanisms of action include causing damage to the membrane and cell wall, increasing unsaturated fatty acid production, disrupting protein metabolism, uncoupling protein translocation, reducing adenosine triphosphate (ATP) levels, inhibiting the Krebs cycle and extracellular toxin secretion, inducing cell cycle arrest, interfering with quorum sensing, and inhibiting biofilm formation.^50^ With regard to this last mechanism, in the present study we also observed that subinhibitory concentrations of BA, both alone and combined with Hs-PPy, were able to inhibit biofilm formation in a strong biofilm-producing MDR *K. pneumoniae* isolate.

Given these findings, combination therapy represents a strategy to combat bacterial resistance, potentially using substances that interfere with biofilm production and act synergistically, making bacteria more accessible to antimicrobial agents.^51,52^ Antimicrobial combinations offer advantages such as reduced toxicity and decreased antimicrobial resistance development, proving more effective compared to single-agent therapy.^51,53^ BA does not exhibit cytotoxicity in cultures of primary nontumor cells (PLP2) and human skin fibroblasts (HSF), nor in tumor cells such as cervical carcinoma (HeLa) and breast adenocarcinoma (MCF-7), presenting an IC50 greater than 400 μg/mL.^50^ Therefore, the BA/Hs-PPy combination has potential for the development of a product for disinfecting surfaces and equipment, aiding in the control of the spread of *K. pneumoniae* on hospital surfaces.

## 5 Conclusions

In conclusion, the *K. pneumoniae* isolates exhibited multidrug resistance and biofilm-forming capacity. Although Hs-PPy showed no intrinsic antimicrobial activity against these isolates, it was able to reduce biofilm formation. BA demonstrated both antimicrobial and antibiofilm activity. The Hs-PPy/BA combination enabled biofilm interference at lower BA concentrations. Therefore, the use of Hs-PPy in combination with BA offers advantages for controlling biofilm formation by MDR *K. pneumoniae*.

## Supplementary Material



## Notes

The origin and identification method of *K. pneumoniae* used in this study; the quantification of biofilm formation and the interference of highly soluble polypyrrole (Hs-PPy) and benzoic acid (BA), alone and in combination (Hs-PPy/BA), against the biofilm of all *K. pneumoniae* isolates in this study (PDF)

## Abbreviations

UNIVASF: Universidade Federal do Vale do São Francisco
ATCC: American type culture collection
BA: Benzoic acid
Hs-PPy: Highly soluble polypyrrole
MIC: Minimum inhibitory concentration
MBC: Minimum bactericidal concentration
MH: Mueller Hinton
BHI: Brain heart infusion
TSB: Trypticase soy broth
OD: Optical density

## References

[1] Haque M., Sartelli M., McKimm J., Bakar M. A. (2018). Health care-associated
infections—an overview. Infect. Drug
Resist..

[2] Braga I. A., Campos P. A., Gontijo-Filho P. P., Ribas R. M. (2018). Multi-hospital point
prevalence study of healthcare-associated infections in 28 adult intensive
care units in Brazil. Journal of Hospital Infection.

[3] Weiner-Lastinger L. M., Abner S., Benin A. L., Edwards J. R., Kallen A. J., Karlsson M., Magill S. S., Pollock D., See I., Soe M. M., Walters M. S., Dudeck M. A. (2020). Antimicrobial-resistant
pathogens associated with pediatric healthcare-associated infections:
summary of data reported to the National Healthcare Safety Network,
2015–2017. Infect. Control Hosp. Epidemiol..

[4] Wyres K. L., Holt K. E. (2018). Klebsiella pneumoniae as a key trafficker
of drug resistance
genes from environmental to clinically important bacteria. Curr. Opin. Microbiol..

[5] Mencarelli J. M., Costa B. E., Gonçalves G. C., Faria A. G., de Souza A. C., Oliveira C. D. M. S., Rufino L. R. A. (2021). Prevalência de klebsiella
pneumoniae em cães e seus tutores. Res.
Soc. Develop..

[6] Pendleton J. N., Gorman S. P., Gilmore B. F. (2013). Clinical
relevance of the ESKAPE
pathogens. Expert review of anti-infective therapy.

[7] De
Oliveira D. M., Forde B. M., Kidd T. J., Harris P. N., Schembri M. A., Beatson S. A., Walker M. J. (2020). Antimicrobial resistance
in ESKAPE pathogens. Clin. Microbiol. Rev..

[8] Salam M. A., Al-Amin M. Y., Salam M. T., Pawar J. S., Akhter N., Rabaan A. A., Alqumber M. A. (2023). Antimicrobial resistance:
a growing
serious threat for global public health. In
Healthcare.

[9] Wang G., Zhao G., Chao X., Xie L., Wang H. (2020). The characteristic
of virulence, biofilm and antibiotic resistance of Klebsiella pneumoniae. International journal of environmental research and public
health.

[10] McCourt J., O’Halloran D. P., McCarthy H., O’Gara J. P., Geoghegan J. A. (2014). Fibronectin-binding
proteins are required for biofilm
formation by community-associated methicillin-resistant Staphylococcus
aureus strain LAC. FEMS microbiology letters.

[11] Shadkam S., Goli H. R., Mirzaei B., Gholami M., Ahanjan M. (2021). Correlation
between antimicrobial resistance and biofilm formation capability
among Klebsiella pneumoniae strains isolated from hospitalized patients
in Iran. Annals of Clinical Microbiology and
Antimicrobials.

[12] Silva N. B. S., Alves P. G. V., de Andrade M. L., Silva S. F., de Oliveira F. G., de Araújo L. B., de Brito Röder D. V. D. (2021). Quantification
of biofilm produced by clinical, environment and hands’ isolates
Klebsiella species using colorimetric and classical methods. J. Microbiol. Methods.

[13] Haque M., McKimm J., Sartelli M., Dhingra S., Labricciosa F. M., Islam S., Charan J. (2020). Strategies
to prevent healthcare-associated
infections: a narrative overview. Risk Manage.
Healthcare Policy.

[14] Ajiboye T. O., Skiebe E., Wilharm G. (2018). Phenolic acids
potentiate colistin-mediated
killing of Acinetobacter baumannii by inducing redox imbalance. Biomed. Pharmacother..

[15] Del
Olmo A., Calzada J., Nuñez M. (2017). Benzoic acid and its derivatives
as naturally occurring compounds in foods and as additives: Uses,
exposure, and controversy. Critical reviews
in food science and nutrition.

[16] Oliveira P. H. R., Reis R. R., Acid B. (2017). Ácido Benzóico
(CAS
65-85-0). Rev. Virt. Quím..

[17] Garcez D. C. P., Ribeiro G., Kominkiewicz M., da Costa M. M., Chideroli R. T., Rosa D. S., Girardini L. K. (2023). Synergy
between polypyrrol and benzoic
acid against antibiotic-resistant Salmonella spp. J. Appl. Microbiol..

[18] da
Silva F. A., Queiroz J. C., Macedo E. R., Fernandes A. W., Freire N. B., da Costa M. M., de Oliveira H. P. (2016). Antibacterial
behavior of polypyrrole: The influence of morphology and additives
incorporation. Mater. Sci. Eng., C.

[19] Clinical and Laboratory Standards Institute (CLSI) Methods for Dilution Antimicrobial Susceptibility Tests for Bacteria that Grow Aerobically, 11th ed. CLSI Standard M07: Wayne, 2023

[20] Krumperman P. H. (1983). Multiple
antibiotic resistance indexing of Escherichia coli to identify high-risk
sources of fecal contamination of foods. Applied
and environmental microbiology.

[21] Kang T. S., Lee S. W., Joo J., Lee J. Y. (2005). Electrically
conducting
polypyrrole fibers spun by electrospinning. Synth. Met..

[22] CLSI . Methods for Dilution Antimicrobial Susceptibility Tests for Bacteria That Grow Aerobically, 11th ed. Clinical and Laboratory Standards Institute: Wayne, PA, 2018. CLSI standard M07.

[23] Stepanović S., Vuković D., Hola V., Bonaventura G. D., Djukić S., Ćirković I., Ruzicka F. (2007). Quantification
of biofilm in microtiter plates: overview of testing conditions and
practical recommendations for assessment of biofilm production by
staphylococci. Apmis.

[24] Merino N., Toledo-Arana A., Vergara-Irigaray M., Valle J., Solano C., Calvo E., Lasa I. (2009). Protein A-mediated multicellular
behavior in Staphylococcus aureus. J. Bacteriol..

[25] Stepanović S., Vuković D., Dakić I., Savić B., Švabić-Vlahović M. (2000). A modified
microtiter-plate test
for quantification of staphylococcal biofilm formation. J. Microbiol. Methods.

[26] Nostro A., Roccaro A. S., Bisignano G., Marino A., Cannatelli M. A., Pizzimenti F. C., Blanco A. R. (2007). Effects of oregano, carvacrol and
thymol on Staphylococcus aureus and Staphylococcus epidermidis biofilms. J. Med. Microbiol..

[27] Wang X., Yao X., Zhu Z. A., Tang T., Dai K., Sadovskaya I., Jabbouri S. (2009). Effect of berberine on Staphylococcus epidermidis biofilm
formation. Int. J. Antimicrob. Agents.

[28] Piepenbrock E., Higgins P. G., Wille J., Xanthopoulou K., Zweigner J., Jahn P., Seifert H. (2020). Klebsiella
variicola
causing nosocomial transmission among neonates—an emerging
pathogen?. J. Med. Microbiol..

[29] Anes J., Hurley D., Martins M., Fanning S. (2017). Exploring the genome
and phenotype of multi-drug resistant Klebsiella pneumoniae of clinical
origin. Frontiers in microbiology.

[30] Mohd
Asri N. A., Ahmad S., Mohamud R., Mohd Hanafi N., Mohd Zaidi N. F., Irekeola A. A., Yusof N. Y. (2021). Global prevalence
of nosocomial multidrug-resistant Klebsiella pneumoniae: a systematic
review and meta-analysis. Antibiotics.

[31] Meletis G. (2016). Carbapenem
resistance: overview of the problem and future perspectives. Therapeutic advances in infectious disease.

[32] Pennini M. E., De Marco A., Pelletier M., Bonnell J., Cvitkovic R., Beltramello M., Stover C. K. (2017). Immune stealth-driven O2 serotype
prevalence and potential for therapeutic antibodies against multidrug
resistant Klebsiella pneumoniae. Nat. Commun..

[33] Rodrigues D., Baldissera G. S., Mathos D., Sartori A., Zavascki A. P., Rigatto M. H. (2021). Amikacin for the treatment of carbapenem-resistant
Klebsiella pneumoniae infections: clinical efficacy and toxicity.
Brazilian Jo4rnal of. Microbiology.

[34] Neuner E. A., Gallagher J. C. (2017). Pharmacodynamic
and pharmacokinetic considerations
in the treatment of critically Ill patients infected with carbapenem-resistant
Enterobacteriaceae. Virulence.

[35] Zavascki A. P., Klee B. O., Bulitta J. B. (2017). Aminoglycosides
against carbapenem-resistant
Enterobacteriaceae in the critically ill: the pitfalls of aminoglycoside
susceptibility. Expert review of anti-infective
therapy.

[36] Rodrigues D., Baldissera G. S., Mathos D., Sartori A., Zavascki A. P., Rigatto M. H. (2021). Amikacin for the treatment of carbapenem-resistant
Klebsiella pneumoniae infections: clinical efficacy and toxicity. Brazilian Journal of Microbiology.

[37] Varesano A., Vineis C., Aluigi A., Rombaldoni F., Tonetti C., Mazzuchetti G. (2013). Antibacterial
efficacy of polypyrrole
in textile applications. Fibers Polym..

[38] Ramirez D. O. S., Varesano A., Carletto R. A., Vineis C., Perelshtein I., Natan M., Gedanken A. (2019). Antibacterial
properties of polypyrrole-treated
fabrics by ultrasound deposition. Mater. Sci.
Eng., C.

[39] Comstock L. E., Kasper D. L. (2006). Bacterial glycans:
key mediators of diverse host immune
responses. Cell.

[40] Rendueles O., Garcia-Garcerà M., Néron B., Touchon M., Rocha E. P. (2017). Abundance and co-occurrence
of extracellular
capsules increase environmental breadth: Implications for the emergence
of pathogens. PLoS pathogens.

[41] Pan Y. J., Lin T. L., Chen C. T., Chen Y. Y., Hsieh P. F., Hsu C. R., Wang J. T. (2015). Genetic
analysis of capsular polysaccharide
synthesis gene clusters in 79 capsular types of Klebsiella spp. Sci. Rep..

[42] Walker K. A., Miller V. L. (2020). The intersection
of capsule gene expression, hypermucoviscosity
and hypervirulence in Klebsiella pneumoniae. Curr. Opin. Microbiol..

[43] Clements A., Gaboriaud F., Duval J. F., Farn J. L., Jenney A. W., Lithgow T., Strugnell R. A. (2008). The major
surface-associated saccharides
of Klebsiella pneumoniae contribute to host cell association. PLoS One.

[44] Jenney A. W., Clements A., Farn J. L., Wijburg O. L., McGlinchey A., Spelman D. W., Strugnell R. A. (2006). Seroepidemiology
of Klebsiella pneumoniae
in an Australian Tertiary Hospital and its implications for vaccine
development. J. Clin. Microbiol..

[45] Singh S., Wilksch J. J., Dunstan R. A., Mularski A., Wang N., Hocking D., Strugnell R. A. (2022). LPS O antigen
plays a key role in
Klebsiella pneumoniae capsule retention. Microbiol.
Spectrum.

[46] Khan F., Manivasagan P., Pham D. T. N., Oh J., Kim S. K., Kim Y. M. (2019). Antibiofilm
and antivirulence properties of chitosan-polypyrrole
nanocomposites to Pseudomonas aeruginosa. Microbial
pathogenesis.

[47] Begini F., Krasowska D., Jasiak A., Drabowicz J., Santi C., Sancineto L. (2020). Continuous flow synthesis of 2, 2′-diselenobis
(benzoic acid) and derivatives. Reaction Chemistry
& Engineering.

[48] Kronenberger T., Ferreira G. M., de Souza A. D. F., da Silva S. S., Poso A., Ribeiro J. A., Parise-Filho R. (2020). Design, synthesis and biological
activity of novel substituted 3-benzoic acid derivatives as MtDHFR
inhibitors. Bioorg. Med. Chem..

[49] Sharma P., Kakkar S., Khatkar A. (2020). Synthesis and Urease
Inhibition Activity
of 4-hydroxy-3-methoxy benzoic acid Derivatives. Res. J. Pharm. Technol..

[50] El-Zawawy N. A., Ali S. S., Khalil M. A., Sun J., Nouh H. S. (2022). Exploring
the potential of benzoic acid derived from the endophytic fungus strain
Neurospora crassa SSN01 as a promising antimicrobial agent in wound
healing. Microbiological Research.

[51] Maione A., La Pietra A., de Alteriis E., Mileo A., De Falco M., Guida M., Galdiero E. (2022). Effect of myrtenol and its synergistic
interactions with antimicrobial drugs in the inhibition of single
and mixed biofilms of candida auris and klebsiella pneumoniae. Microorganisms.

[52] Vaidya M. Y., McBain A. J., Butler J. A., Banks C. E., Whitehead K. A. (2017). Antimicrobial
efficacy and synergy of metal ions against Enterococcus faecium, Klebsiella
pneumoniae and Acinetobacter baumannii in planktonic and biofilm phenotypes. Sci. Rep..

[53] Bisso
Ndezo B., Tokam Kuaté C.
R., Dzoyem J. P. (2021). Synergistic
antibiofilm efficacy of thymol and piperine in combination with three
aminoglycoside antibiotics against Klebsiella pneumoniae biofilms. Can. J. Infect. Dis. Med. Microbiol..

