# Supplementary material for: Antimicrobial and Antibiofilm Activities of Highly Soluble Polypyrrole Combined with Benzoic Acid against Multidrug-Resistant Klebsiella pneumoniae
Source: ACS Omega. 2025 Nov 24;10(48):59200–7. doi: 10.1021/acsomega.5c08466 (PMC12771181; doi:10.1021/acsomega.5c08466)
Supplement: Supplementary file 1 [file ao5c08466_si_001.pdf]

Antimicrobial and antibiofilm activity of highly soluble polypyrrole combined with benzoic acid against multidrug-resistant *Klebsiella pneumoniae*

Danillo S. Rosa [a], *Brendo A. dos S. Da Cruz* [b], *Nayara Andreo* [b], *Betilde de M. Silva* [b], *Priscila I. de Figueirêdo* [b], *Fernando Antônio Gomes da Silva Júnior* [b], *Helinando Pequeno de Oliveira* [b], *Flavia Figueira Aburjaile* [c], *Carine Rosa Naue* [d], *Mateus Matiuzzi da Costa* [b]*

[a] Universidade Federal Rural de Pernambuco, Rua Dom Manuel de Medeiros, s/n, Dois Irmãos, Recife, Pernambuco 52171 900, Brazil

[b] Universidade Federal do Vale do São Francisco (UNIVASF), Avenida José de Sá Maniçoba, s/n, Centro, Petrolina, Pernambuco 56300-000, Brazil

[c] Universidade Federal de Minas Gerais, Avenida Presidente Antonio Carlos, 6627, Pampulha, Belo Horizonte, Minas Gerais, 31270-901, Brazil

[d] Hospital Universitário da Universidade Federal do Vale do São Francisco, Avenida José de Sá Maniçoba, s/n, Centro, Petrolina, Pernambuco 56304-205, Brazil

*mmatiuzzicosta@gmail.com

Supplementary Material

Table S1 Origin and identification method of *Klebsiella pneumoniae* used in this study

Number	Source	Isolation source	Identification method
253	HU-UNIVASF	Blood culture	Phoenix equipment
3042	HU-UNIVASF	Rectal swab	Phoenix equipment
3045	HU-UNIVASF	Rectal swab	Phoenix equipment
3065	HU-UNIVASF	Rectal swab	Phoenix equipment
3099	HU-UNIVASF	Surveillance culture	Phoenix equipment
3105	HU-UNIVASF	Tracheal secretion	Phoenix equipment
3109	HU-UNIVASF	Surveillance culture	Phoenix equipment
3116	HU-UNIVASF	Surveillance culture	Phoenix equipment
3122	HU-UNIVASF	Surveillance culture	Phoenix equipment
3125	HU-UNIVASF	Surveillance culture	Phoenix equipment
3126	HU-UNIVASF	Rectal swab	Phoenix equipment
3130	HU-UNIVASF	Rectal swab	Phoenix equipment
3132	HU-UNIVASF	Urine culture	Phoenix equipment
3139	HU-UNIVASF	Rectal swab	Phoenix equipment
3164	HU-UNIVASF	Rectal swab	Phoenix equipment
3172	HU-UNIVASF	Rectal swab	Phoenix equipment
3174	HU-UNIVASF	Rectal swab	Phoenix equipment
3179	HU-UNIVASF	Rectal swab	Phoenix equipment
3193	HU-UNIVASF	Rectal swab	Phoenix equipment
3233	HU-UNIVASF	Rectal swab	Phoenix equipment
3271	HU-UNIVASF	Rectal swab	Phoenix equipment
3272	HU-UNIVASF	Rectal swab	Phoenix equipment
3276	HU-UNIVASF	Rectal swab	Phoenix equipment
3278	HU-UNIVASF	Rectal swab	Phoenix equipment
3286	HU-UNIVASF	Rectal swab	Phoenix equipment
5733	HU-UNIVASF	Brain abscess capsule	Phoenix equipment
5735	HU-UNIVASF	Urethral swab	Phoenix equipment
5773	HU-UNIVASF	Tracheal secretion	Phoenix equipment
5794	HU-UNIVASF	Tracheal secretion	Phoenix equipment
5799	HU-UNIVASF	Tracheal secretion	Phoenix equipment
5813	HU-UNIVASF	Tracheal secretion	Phoenix equipment
5825	HU-UNIVASF	Soft parts	Phoenix equipment
5836	HU-UNIVASF	Rectal swab	Phoenix equipment
5887	HU-UNIVASF	Tracheal secretion	Phoenix equipment
5887.2	HU-UNIVASF	Tracheal secretion	Phoenix equipment
5893	HU-UNIVASF	Tracheal secretion	Phoenix equipment
5984	HU-UNIVASF	Tracheal secretion	Phoenix equipment
5994	HU-UNIVASF	Tracheal secretion	Phoenix equipment
6001	HU-UNIVASF	Tracheal secretion	Phoenix equipment
6002	HU-UNIVASF	Tracheal secretion	Phoenix equipment
9123	HU-UNIVASF	Urine culture	Phoenix equipment
9264	HU-UNIVASF	Surveillance culture	Phoenix equipment
9276	HU-UNIVASF	Urine culture	Phoenix equipment
9309	HU-UNIVASF	Urine culture	Phoenix equipment
9322	HU-UNIVASF	Urine culture	Phoenix equipment
9338	HU-UNIVASF	Urine culture	Phoenix equipment
9944	HU-UNIVASF	Blood culture	Phoenix equipment

HU-UNIVASF: Hospital Universitário da Universidade Federal do Vale do São Francisco (University Hospital of Federal University of the Vale do São Francisco).

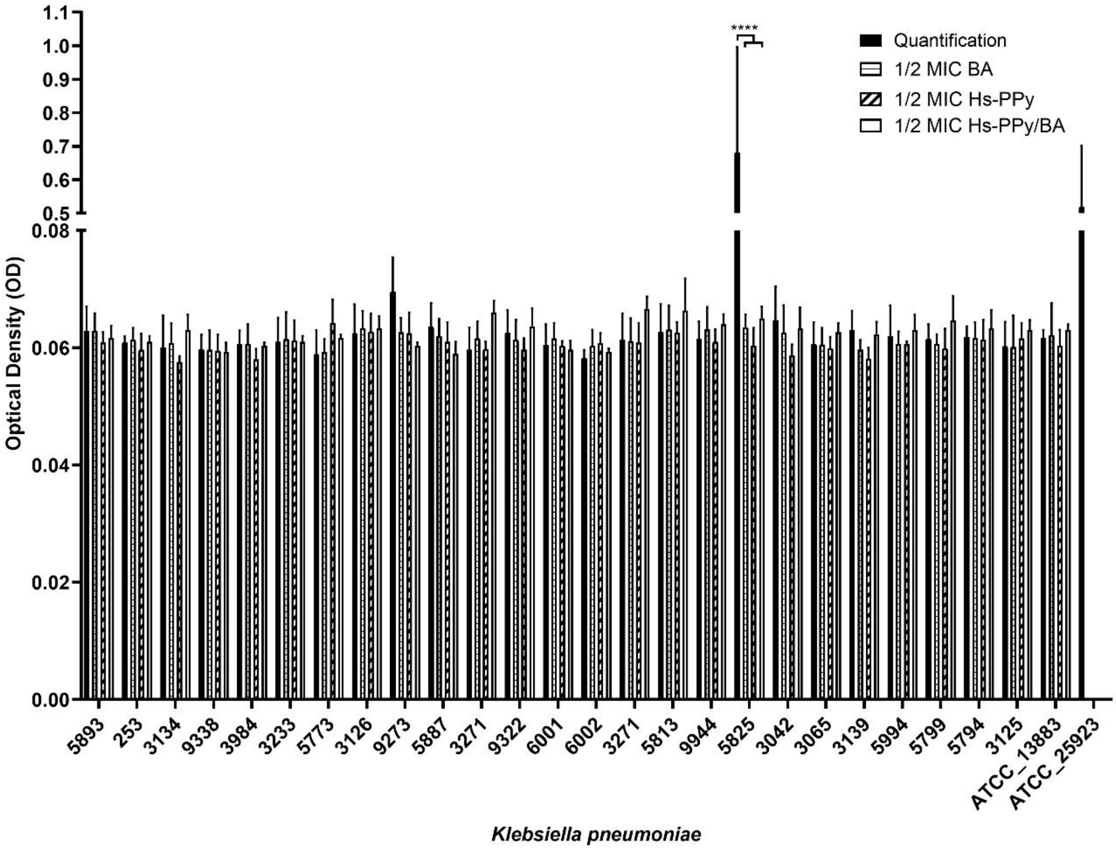

Figure S1. Quantification of biofilm formation and interference by highly soluble polypyrrole (Hs-PPy) and benzoic acid (BA), alone and combined (Hs-PPy/BA), against biofilm of all *Klebsiella pneumoniae* isolates in the present study. ATCC_13883: *K. pneumoniae* reference strain; ATCC_25923: Methicillin-sensitive S. aureus (biofilm-positive control). Negative control OD620: 0.057±0.003 (not subtracted from analyses). ****$p < 0.0001$.
